# Building Principles for Constructing a Mammalian Blastocyst Embryo

**DOI:** 10.3390/biology7030041

**Published:** 2018-07-23

**Authors:** Peter L. Pfeffer

**Affiliations:** School of Biological Sciences, Victoria University of Wellington, Wellington 6012, New Zealand; peter.pfeffer@vuw.ac.nz; Tel.: +64-4-463-7462

**Keywords:** lineage determination, patterning, blastomere polarization, compaction, cleavage stages, morula, gene regulatory networks

## Abstract

The self-organisation of a fertilised egg to form a blastocyst structure, which consists of three distinct cell lineages (trophoblast, epiblast and hypoblast) arranged around an off-centre cavity, is unique to mammals. While the starting point (the zygote) and endpoint (the blastocyst) are similar in all mammals, the intervening events have diverged. This review examines and compares the descriptive and functional data surrounding embryonic gene activation, symmetry-breaking, first and second lineage establishment, and fate commitment in a wide range of mammalian orders. The exquisite detail known from mouse embryogenesis, embryonic stem cell studies and the wealth of recent single cell transcriptomic experiments are used to highlight the building principles underlying early mammalian embryonic development.

## 1. Introduction

The German word “Bauplan” (meaning ‘building plan’ or ‘blueprint’) was introduced to the English-speaking community of embryologists by Woodger [1], to describe the archetypical body plan of a particular group of animals. The architectural connotation is useful, as in many ways the formation of an embryo resembles the building of a house: In both, a three-dimensional structure is created from a limited set of building materials, with construction following a set of principles that have been optimised over time for cost, speed and quality. Designs are adapted to local material availability, the environment, and the future uses of the building. For an embryo, the building materials are the different types of cells and extracellular secretions, which appear to have been carefully designed to fit together in very limited ways (the building principles) thanks to highly detailed instructions encoded by a temporal series of gene regulatory networks. The truly amazing part though, is that no “outside” help is required in the building of an embryo. How is this possible? In this first review covering only the initial steps of embryogenesis, I will address the remarkable innovations which evolution has selected to automate the generation of a fairly consistent mammalian late-blastocyst Bauplan, while starting out from a myriad of divergent initial environments.

### 1.1. Mammalian Early Embryological Diversity

**Figure 1:**
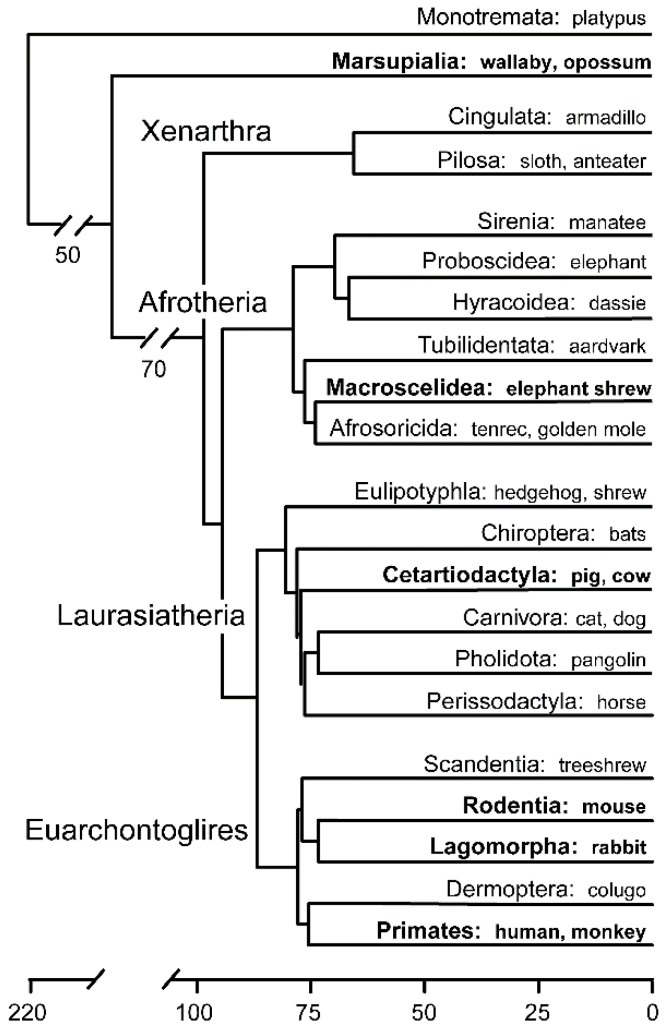
Divergence time (in millions of years) of all extant mammalian orders [2] and their associated superordinal groupings. Groups discussed are shown in bold.

Before delving into mechanistic aspects, a brief description of mammalian taxonomy, early embryogenesis and the bewildering nomenclature (Table 1) is warranted. Class Mammalia consists of eutherian (placental) mammals, monotherian (marsupial) mammals and their sister group, the egg-laying monotremes. Within the evolutionary successful eutherians there are 19 extant orders, divided into four superordinal groups, and then four subgroups: Euarchontoglires (e.g., primates, rodents, rabbits), Laurasiatheria (e.g., carnivores, ruminants, pigs, horses, bats, whales), Afrotheria (e.g., elephants, sea cows, tenrecs) and Xenarthra (e.g., armadillos, anteaters)—all of which last shared a common ancestor about 100 million years ago (Figure 1) [2]. Before the molecular era, the embryology of representatives of numerous of these orders were examined [3], however during the last 40 years, investigations increasingly focused on only a couple of species, most prominently the easily kept laboratory animals (mouse, rat, rabbit), with some additional work done on farm animals.

Excitingly, with new molecular technologies, the wealth of mechanistic insight into development that could previously only be obtained from the mouse model, is being applied to re-investigate embryos from other mammalian orders, opening a new phase of comparative molecular embryology that is revealing (somewhat ironically) that early mouse development is atypical of most mammals.

### 1.2. Morphological Events Leading to the Blastocyst

**Figure 2:**
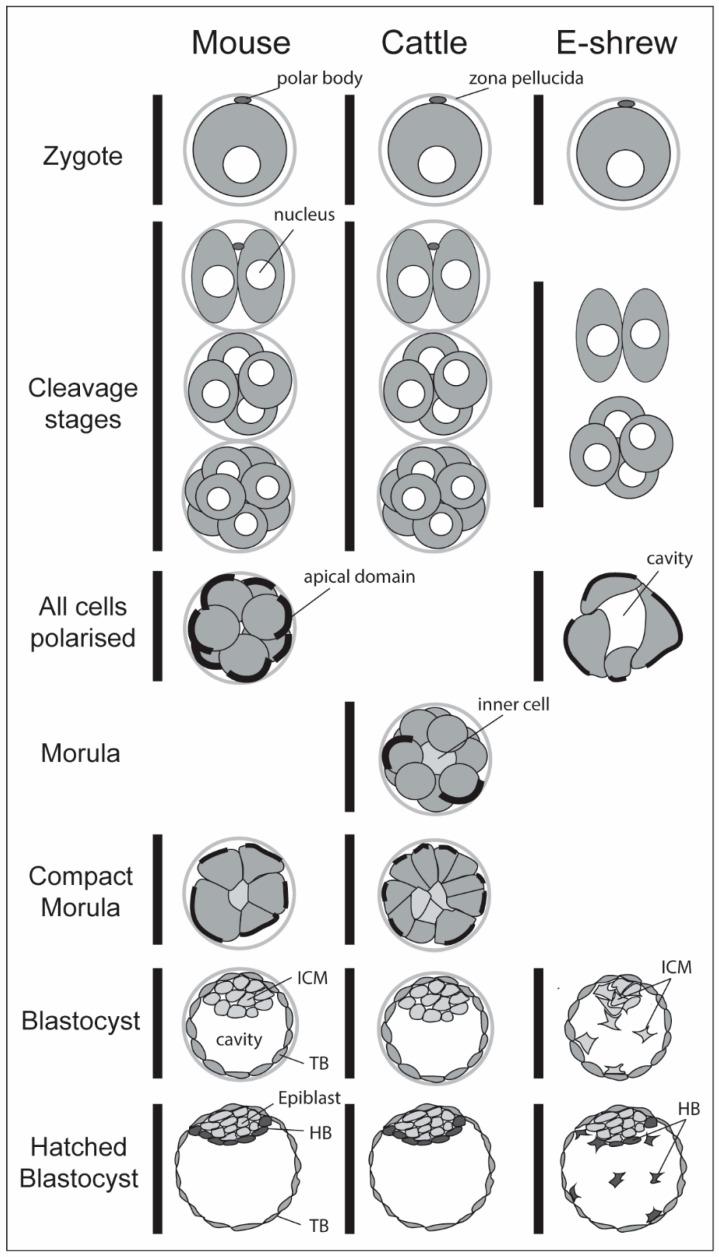
Main developmental stages in an Euarchontoglirian (mouse), Laurasiatherian (cattle) and Afrotherian (elephant shrew). While the sequence of morphological events is largely conserved, some species lack certain stages: mouse embryos compact already at the 8-cell (cleavage) stage, thus skipping the un-compacted morula stage; cattle embryos do not pass through a stage where all blastomeres are polarised and elephant shrews miss out on morula stages altogether and lose their zona pellucida prematurely, immediately after the 1-cell stage. The cattle sequence also applies to the Euarchontoglirian rabbit and human embryo. HB; hypoblast; ICM, inner cell mass; TB, trophoblast.

Eutherian mammalian development (Figure 2) commences after fertilisation, when the zygote undergoes several cleavages without concomitant growth. The resultant blastomeres are contained by a proteinaceous zona pellucida shell. In Laurasiatherians and Euarchontoglirans (which together constitute most mammals), the blastomeres compact together to form a morula with the outermost cells forming an epithelium. The innermost cells are apolar, and their number increases by cell division and contributions from the outside layer. Eventually a cavity develops (the “blastocyst cavity”, also referred to as blastocoel), marking the onset of the blastocyst stage, characterised by an outside layer of polarised cells termed the trophoblast or trophectoderm and a clump of inner cells called the inner cell mass (ICM). In contrast, in Afrotherians of the orders Macroscelidea (elephant shrew) and Afrosoricida (tenrec), the morula stage is bypassed (Figure 2), with blastomeres first forming a hollow unilaminar vesicle, from which future ICM cells bud off, as in the elephant shrew [4], or in which future ICM cells proliferate in one region and somehow relocate to the inside of the vesicle as in tenrecs [5]. The net result in all cases is a locally bilayered structure with trophoblast, which faces the maternal environment on the outside, covering the ICM cells located asymmetrically on the inside. Note that in marsupial mammals a trophoblast/ICM bilayer is not formed, instead, a large patch of the unilaminar vesicle becomes the pluriblast with a fate equivalent to the eutherian ICM [6]. The trophoblast will contribute to the foetal part of the placenta but not to the foetus (embryo proper). The next morphological event is the segregation of hypoblast cells, which form a unicellular layer beneath the ICM. From this point in development the ICM cells that have not contributed to the hypoblast are referred to as epiblast (epi). While not discussed in this review, hypoblast cells (the “third lineage”, see Section 5) will eventually give rise mainly to extraembryonic components of the conceptus. Such as, the inner layer of the yolk sac and, in primates, extraembryonic mesenchyme which, together with epiblast-derived extraembryonic mesoderm forms most of the umbilical cord and the connective tissue and blood vessels supporting extraembryonic membranes. Recent evidence from the mouse though has also indicated contribution of hypoblast to the definitive endoderm [7]. The epiblast forms all parts of the embryo proper as well as contributing to extraembryonic tissues such as the amnion, allantois and extraembryonic mesoderm.

## 2. Gearing up for Autonomy (Cleavage Stages)

One of the most difficult challenges for multicellular animals is the maternal to embryonic transition, which involves the activation of the newly conjoined genetic material so as to drive the embryonic gene program, while at the same time maintaining basic cellular needs using the stores of protein and RNA inherited from the mother via the egg. Embryonic gene activation (EGA, also called zygotic genome activation) occurs in mammals during the cleavage stages in successive “waves” or phases of gene set activations of increasing magnitude. The major phase, involving the switching on of over a thousand genes, happens at the late 2-cell stage in mice, at 4 cells in rabbits, 4–8 cells in humans and pigs and 8–16 cells in cattle [8]. How is this brought about? Two non-exclusive mechanisms are conceivable. (1) An external trigger, as supplied by the sperm or from the maternal reproductive tract, kick-starts EGA; (2) an internal time delay mechanism operates, involving the gradual decay/dilution of inhibitors and/or the activation of “pioneer” factors stored in the egg.

### 2.1. Triggering EGA Via an External Signal

The fact that eggs (i) can be activated to undergo EGA and further development without sperm; and (ii) can be fertilised and grown outside the maternal environment, argues against the importance of external stimuli. However, very recently the Banerjee lab found an extraordinary link between EGA and pyruvate availability [9]. It has long been known that pyruvate is preferred to glucose as energy substrate for mammalian cleavage-stage embryos [10], as their low energy requirements results in a high ATP:ADP ratio which inhibits phosphofructokinase, the gatekeeper for glucose glycolysis [11]. Nagaraj and co-workers now showed that prior to EGA, pyruvate is required to translocate the mitochondrial enzymes involved in the first half of the tricarboxylic acid (TCA) cycle into the nucleus! Removal of pyruvate blocks the embryo before EGA and prevents histone modifications associated with the opening up of chromatin (H3K4 and H3K27 acetylation; H3K27 trimethylation), due to a reduction of the metabolites required for these modifications [9]. This mechanism appears to be conserved: in humans, pyruvate dehydrogenase, required for Acetyl-CoA production, is found in the nucleus only from the 4 to 8 cell stages, concomitant with EGA in this species [9]. Thus, pyruvate availability in the oviduct regulated by the mother in a time- or location-dependent fashion (the embryo is propelled along the oviduct toward the uterus during cleavage stages), may function as an extrinsic mechanism that, even if not sufficient, is necessary for EGA activation.

### 2.2. Intrinsic Embryonic Genome Activation

**Figure 3:**
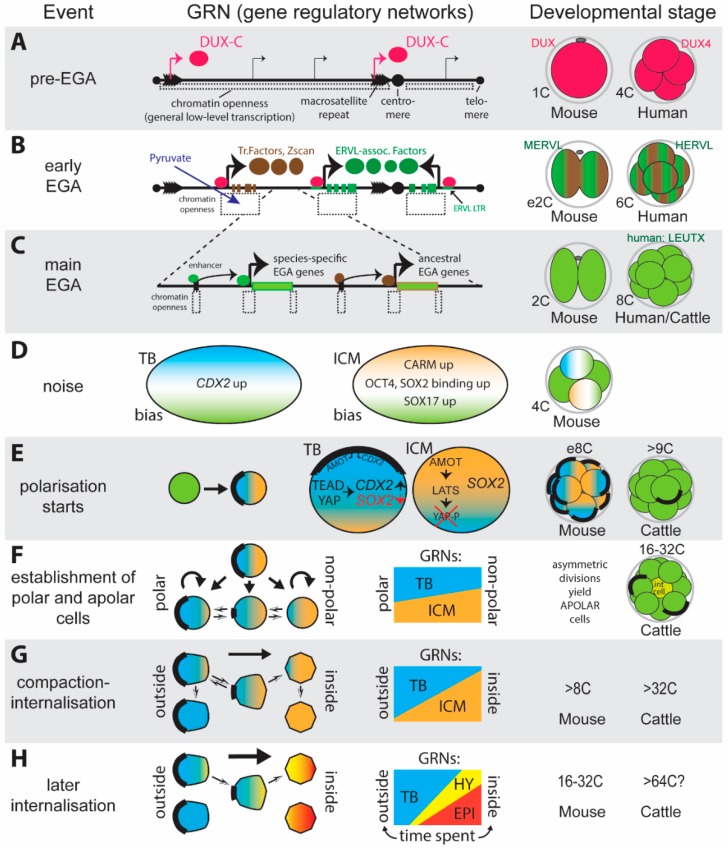
**A summary of pre-blastocyst mammalian development with a focus on gene expression.** (**A**) Post-fertilisation but prior to embryonic gene activation (EGA) a loosening of chromatin leads to transcription of the highly repetitive *DUX-C* genes found in macrosatellite regions. (**B**) DUX-C protein binding leads to further chromatin opening and transcription of early-EGA target genes. This may require exogenous pyruvate-dependent translocation of mitochondrial TCA enzymes into the nucleus. Binding of the more divergent first homeodomain of DUX-C occurs particularly in the LTR of ERVL-like retrotransposons and leads to species-specific transcription of EGA factors. Binding via the second DUX-C homeodomain in turn activates sets of evolutionarily conserved EGA genes. (**C**) During main EGA (shown in green), transcription factors previously activated by DUX-C, such as LEUTX in humans, lead to EGA in an increasingly restrictive chromatin environment. (**D**) Levels or binding kinetics of lineage specific transcription factor protein or transcripts, activated during EGA, show regional heterogeneity due to stochastic (noise) events and leads to a potential bias in lineage, shown by blue for trophoblast and orange for inner cell mass. (**E**) Formation of apical domains (AD) leads to asymmetry within blastomeres due to tethering of *Cdx2* RNA and the YAP-inactivator AMOT. Cells with an AD thus are biased towards a trophoblast fate. (**F**) In mice, the default state is polarisation: non-polar cells are generated via asymmetrical division. Cells with less AD are biased toward the ICM lineage. In other mammals the default state is non-polar and blastomeres gradually acquire polarity. A relationship between presence of an AD and lineage bias has not yet been examined. (**G**) Compaction aids the internalisation of non-polar cells to the inside of the embryo. A strong lineage bias is seen. (**H**) Cells internalised earlier appear to be biased toward the epiblast lineage, those later to the hypoblast lineage. At this stage numerous cells are committed to either TB or ICM-derived lineages while the hypoblast-epiblast lineages within ICM progenitor cells are not yet resolved.

Does an internal delay mechanism operate as well, such as is seen in the fruit fly *Drosophila*, where the maternally encoded zinc-finger protein Zelda has been identified as the main activator of the embryonic genome [12]? It appears mammals may also have a master EGA activator (Figure 3A), in the form of the double-homeodomain-containing DUX-C protein [13,14,15]. Both the human DUX-C homologue, termed DUX4, and the mouse DUX-C homologue, termed DUX, are expressed before the first EGA phase in each species. DUX4/DUX overexpression in embryonic stem cells induces hundreds of genes known to be upregulated at the start of EGA. This gene activation was shown to be associated with the opening up of chromatin specifically around EGA target genes, concomitant with direct DUX binding [15]. Lastly, embryonic DUX depletion leads to defective EGA in mouse embryos [14]. Two features are likely to have contributed to the selection of DUX-C orthologues as the instigators of EGA. First, the increased and potentially synergistic binding provided by two separate DNA binding domains within one protein are likely to make this transcription factor a pioneer factor, and indeed DUX4 was shown to be able to bind at DNaseI inaccessible sites and, via p300/CBP recruitment, to acetylate histone H3 at lysine 27 to open up chromatin [16]. Secondly, the *DUX4* gene is found in tandem repeats within individual units of macrosatellite repeat regions, making it the highest copy-number protein-coding gene within the human genome. Indeed, *DUX-C*-family homologues (*DUX-C*, *DUX4*, *Dux*) in species from all four mammalian superordinal groups show a macrosatellite tandem-array organisation with very high copy numbers [17]. Thus, even a modest transcriptional activation of each gene copy will result in an overall massive burst of protein, able to drive a crucial event such as the activation of the genome. The *DUX-C*-containing macrosatellites are found either close to the telomere ends (or in mice, a chromosomal fusion point marking a prior telomere end) or close to the centromere (pericentrosomic) [17]. Regions generally form constitutive heterochromatin, which is kept in a transcriptionally tightly repressed state via distinct epigenetic mechanisms [18]. So how are the *DUX-C* genes then activated? It has been shown that upon fertilisation the mouse egg enters a globally transcriptionally permissive state characterised by (i) very extensive low-level transcription even in intergenic and repetitive regions and (ii) an independence of enhancer elements with spurious transcriptional initiation occurring at cryptic promoters [19]. This permissive state is likely driven by a loosening of chromatin, as demonstrated by ATAC-sequencing [20] and the detection of increased histone mobility [21] seen specifically at the mouse zygote stage. Whether such low-level ubiquitous transcription, in combination with the now accessible and extremely high abundance of *DUX-C* genes, is sufficient to generate sufficient DUX-C protein to initiate its downstream effects, remains to be determined.

Comparing human DUX4- and mouse DUX-activated genes in human as well as mouse cells, revealed the existence of two sets of target genes that overlapped extensively with genes activated during EGA [13,14,15]. One set, recognised by the more conserved (second) DUX4/DUX homeodomain, was recognised by both DUX4 and DUX, as well as by the dog DUX-C homologue. The second set of target genes was specific to each species with many downstream of included distinct retroviral repeat elements: Human DUX4 strongly activated the HERVL, whereas mouse Dux activated the MERVL-associated genes [13]. It had previously been discovered that many critical EGA genes are under the control of ERVL long terminal repeat enhancers [22]. Now it became evident that many of these enhancers are driven by DUX-C proteins. The picture that thus emerges (Figure 3B,C) is one where all DUX-C family members recognise targets that form the core of an ancestral EGA network. Whereas, EGA genes that have subsequently come under the control of particular retrotransposon classes represent species-specific refinements. The binding and transcriptional activation by DUX-C locally counteracts the increasingly repressive chromatin state that spreads after the brief transcriptionally permissive period, thereby contributing to the establishment of large (median length 40 kbp) stretches of open chromatin during an “early” or “minor” EGA wave [15,20]. These open regions typically are found downstream of DUX-bound, transcribed ERVL elements which drive the expression of early EGA genes. The early EGA genes presumably include transcription factors initiating the “major” wave of EGA. In the mouse major EGA was shown to involve a novel chromatin signature characterised by open chromatin centred over (i) promoters; (ii) distal stage-specific enhancers; and (iii) transcriptional end sites [20]. In humans, one of the early EGA specific genes activated via ERVL-driven transcription is LEUTX, a paired–type homeodomain-containing transcription factor that was shown to activate thousands of genes, including the repressor DPRX, which binds similar targets to LEUTX thereby restricting the extent of EGA [23]. Interestingly no mouse LEUTX homologue exists [23], indicating that these early events have diverged considerably, in line with the use of species-specific DUX-C activated ERVL elements and the different timing of EGA.

### 2.3. Achieving Totipotency

While the EGA gene regulatory networks may show only limited overall resemblance between different mammals, certain critical genes (the ancestral set) may need to be activated by all mammals [24]. One such gene is *Zscan4*, which was shown to be activated in mice and humans by DUX4/Dux [15]. *Zscan4* is required to ensure recombination-mediated telomere extension, which is necessary to rejuvenate and preserve the chromosome ends that are eroded during gametogenesis [25,26]. Most importantly though, is the EGA dependent activation of the master regulatory genes that set up the blastomeres to be able to generate every cell of the conceptus (foetus and extraembryonic/foetal membranes), that is, to be “totipotent”.

Totipotency of peri-EGA embryos was first dramatically shown in rabbits [27] and mice [28], when, after ablation of one blastomere of the 2-cell embryo, the remaining blastomere could result in normal young. In sheep (a Laurasiatherian), four lambs were born by separating the blastomeres from one 4-cell embryo and transferring these cells, embedded in agarose blocks into separate recipient ewes [29,30]. Even in humans, related experiments have been executed, showing that individual blastomeres from a 4-cell embryo are able to reform four entire blastocysts [31].

Yet at some point post-EGA, totipotency begins to be eroded as master lineage regulators compete with each other to eventually establish lineage specific gene regulatory networks (GRN) that stably restrict the fate of subpopulations of embryonic cells. Using a variety of transplantation, cell-mixing and embryo-chimera experiments, it has been shown that the fate of individual cells gradually becomes specified and then stably set (“committed”) along fixed developmental trajectories, at species-specific times (reviewed previously [32]). An exciting question, dealt with in the next section, is how this specification and commitment is achieved, and how well these processes are conserved across different mammalian orders.

In summary, the embryo acquires autonomy post-fertilisation under the permissive influence of externally supplied pyruvate, entering a state of transcriptional promiscuity that leads to the initial activation of pioneering factors such as the multicopy DUX-C family, which instigate a minor wave of embryonic gene activation. Numerous of these early EGA genes are driven by long terminal-repeat enhancers derived from species-specific retrotransposon families, thus allowing their co-ordinate activation. Some of the minor EGA genes, such as LEUTX in humans, lead to major EGA, characterised by the totipotency of the blastomeres.

## 3. Creating Two Environments in the Morula (Inside and Outside)

### 3.1. Establishment of Protein Heterogeneity

How are different stable lineage gene regulatory networks first established when starting from embryonic genome activation that occurs in every blastomere? An important consideration is that EGA is not uniform. Unequal expression levels of genes in sister blastomeres arise with high statistical likelihood, particularly for genes expressed at relatively low levels. Two often concurrent mechanisms contribute to this: Unequal partitioning of cytoplasm (unequal segregation) upon cell division, and the inherent “noise” of transcription, arising because transcription occurs in short but intense bursts [33]. Detailed studies in mice have indicated the early presence of biases in mRNA or protein expression from the 2-cell stage onward. These include differences (i) at the 4-cell stage in the epigenetic modifier PRDM14 [34]; (ii) in CARM1-mediated histone 3 arginine methylation [35]; (iii) in DNA-binding kinetics of the ICM-markers OCT4 (POU5f1) and SOX2 [36,37] and (iv) in mRNA levels of the trophoblast markers *Cdx2* [38] and *Sox21* [39]. Numerous bimodal expression patterns (high in one blastomere, absent in the other), particularly in genes involved in WNT signaling, were reported at the mouse 2-cell stage [40]. Mathematical modelling incorporating segregation effects, transcriptional noise and feedback loops, in combination with single cell expression analyses using mouse and human datasets. This suggests that such early, stochastically arising biases of potential lineage-specifiers are likely to guide, but not fully determine lineage trajectories, as at the 8-cell stage such biases could still be reversed [38]. Thus, it appears that the establishment of heterogeneity is a cell-intrinsic and gradual, progressive process (Figure 3D).

### 3.2. Blastomere Polarisation

While these heterogeneities in gene expression arise, cell division without cell growth results in an increasing number of blastomeres of decreasing size confined within the proteinaceous envelope of the zona pellucida. Blastomeres are initially of rounded appearance, and loosely apposed to each other, but soon acquire polarity, establishing apical domains enriched for microvilli, F-actin and an apical protein-complex containing Pard3,6 and aPKC [41]. In mice, this polarisation process occurs at the 8-cell stage (Figure 3E). It appears to be a cell-intrinsic autonomous event as it occurs in all blastomeres, even when these are kept separated from the 4-cell stage [42]. However, cell-cell contact, mediated either by the ubiquitously expressed E-Cadherin (CADH-1)—or potentially by Cadherin-3 [43], synchronises and speeds up the onset, and directs the axis of polarisation such that the apical domain forms in the centre of the contact-free zone [44]. Importantly, the apical domain attracts one end of the spindle axis thereby strongly biasing the plane of the subsequent cleavage [45]. This can lead to completely asymmetric divisions, where one daughter cell does not inherit any part of the apical domain and thus is apolar.

The embryo thus now consists of two different types of cells—polar and apolar (Figure 3E,F). These cells have measurable differences in key lineage determinants: polar cells express more of the trophoblast lineage determination gene *Cdx2*, and less of the ICM lineage marker *Sox2* [45]. *Cdx2* is a target of TEAD4, a transcription factor that is upregulated by the YAP protein [46]. To be active, YAP has to escape phosphorylation by LATS [47]. This is achieved only in polarised cells, in which the apical domain sequesters Angiomotin [45], thereby preventing it from activating LATS [47,48]. The same pathway, involving active YAP and TEAD4, simultaneously downregulates *Sox2* in these polar cells [49]. Aside from the apical domain/YAP-mediated pathway in transcriptional partitioning of *Cdx2* and *Sox2*, the apical domain is also able to sequester *Cdx2* messenger RNA via a mechanism that involves the apical domain component aPKC and the terminal 97 nucleotides of the *Cdx2* coding region [50].

### 3.3. Blastomere Positioning within the Morula

It follows from this sequence of events that the position of a blastomere within the embryo is important to its eventual fate. As the blastomere number increases, some cells will land up on the inside, surrounded on all sides by other cells. These inside cells are apolar. Why? At the 8-cell stage, when mouse blastomeres acquire polarity, the apical domain will face outwards as this is the only contact-free zone. Due to the subsequent bias for a radially orientated spindle axis, cleavage will be predominantly asymmetric [45,51,52]. In the original polarity model, it was assumed that this would inherently lead to apolar daughter cells situated on the inside [53]. However, time lapse observations revealed significant numbers of apolar cells on the outside [54,55]. Some apolar outside cells, characterised by enhanced YAP activity, secondarily acquire polarity and form an apical domain on their contact-free membranes, while others internalise [54]. This internalisation process is closely linked to compaction. In mice compaction is seen nearly concurrently with polarisation and involves the E-Cadherin mediated adhesion process, whereby blastomeres flatten against each other to form a compacted morula [56]. Compaction creates tension between adjacent cells. Between the 8 and 16 cell stage some of this tension is driven by E-Cadherin-expressing filipodia—at the 8-cell stage half the blastomeres extend these filipodia onto the apical surface of two neighbouring cells for 5 h then retract them during the next hour before the next cell division. Interference with filipodia formation (by knock-down of E-Cadherin, its interaction partners α- and β-Catenin or Myosin-X, or by F-actin inhibition) abolished or delayed compaction, while Myosin-X overexpression caused premature compaction at the 6-cell stage [57].

The high tensile forces arising through the compaction process work in concert with constriction occurring at the apical side of those cells that, after division, are positioned closer to the centre. Such apical constriction reduces the apical area and conversely increases the basolateral surface, thereby gradually positioning the cell closer to the embryo centre. Constricting cells, seen predominantly from the 12-cell stage, were shown to be enriched for Mysosin-II [58]. Remarkably, enrichment of cortical myosin coupled to higher contractility could be linked to apolar cells, which thus internalise in preference to the polar cells. Polar cells, by virtue of inheriting the “stiff” apical domain, are better in resisting apical constriction and remain on the outside [59]. Whether the extensive changes in tension and cell-shape accompanying internalisation of cells (so-called “mechanosensing”) controls YAP activity redundantly to the apical-domain mechanism outlined previously, remains to be seen [59,60].

What emerges from these studies in mouse embryos is a self-organising or self-regulative process, whereby distribution of polarity genes in cell-contact free regions leads to asymmetry within each cell. Because the mitotic spindle aligns with the resulting apical domain, asymmetric divisions ensue, generating two types of cells: polar or apolar. Apolar cells have little or no apical domain, which predisposes them to be positioned towards the inside through the tensile forces generated by compaction. The absence of the apical domain concurrently shuts down YAP activity, thus biasing inside polar cells to an ICM-like GRN (Figure 3F). Thus, morphological events set up lineage gene-regulatory networks. What is still unclear, is whether and how these morphological events relate to the early heterogeneities in gene and protein expression and kinetics seen in 2- to 8-cell mouse embryos. Several questions remain to be answered. Do the early biases affect the decision of an 8-cell blastomere to divide symmetrically (thus generating two polar daughter cells) or asymmetrically (thus contributing apolar cell that is likely to form ICM)? Alternatively, do the early biases affect the timing of cell division? Such altered timing would have downstream lineage determining effects as divisions of blastomeres isolated from 8-cell embryos, after compaction has commenced, are more likely to be asymmetric than divisions of pre-compaction blastomeres [51]. Conversely, do the morphologically-induced biases simply reinforce (or annul) earlier gene expression biases? Additive effects may be necessary to drive expression of particular lineage-determining master genes over a critical threshold that allows, via feedforward loops and negative feedback of alternate lineages, the stabilisation of a particular lineage-GRN [61,62].

### 3.4. Polarisation, Compaction and Cell Positioning in Other Mammals

Is the mouse situation characteristic for other mammals? Similar to embryonic genome activation, polarisation lags behind the mouse in cattle and rabbits, occurring at the 9-15-cell stage in cattle and from the 32-cell stage in rabbits [63]. Interestingly though, only around 40% of cattle blastomeres are polarised between the 9- and 74-cell stage (maximally 52%)—similarly, only 46% of rabbit blastomeres are polarised [63]. Thus, unlike mice, in these mammals the *acquisition of polarity*, as opposed to the *loss* of previously acquired polarity (via asymmetric divisions), leads to two morphologically distinct cells (Figure 3E,F). Secondly, compaction in cattle does not occur at the same stage as polarisation, starting only at the 32-cell stage, more than a full cell division later than polarisation [64]. Allocation of cattle blastomeres to the inside was seen to occur both well before compaction (1–2 inner cells in 20% of 16-cell embryos) and after compaction [64]. Thus, blastomere positioning is temporally more loosely coupled to any tensile compaction forces that may actively drive ICM-lineage biased apolar cells to the interior of the embryo. Remarkably, in members of two mammalian orders belonging to Afrotheria (Figure 1), there are no “inside” cells at all initially and no morula stage exists. Instead, blastomeres of the streaked tenrecs of Madagascar (*Hemicentetes semispinosus*; Order Afrosoricida) line up after the second division (4- to 8-cell) along the zona pellucida to form a one-layered (unilaminar) embryo with a central cavity. By the 16-cell stage inner cells have accumulated at one pole of this cavity [5]. Elephant shrews (*Elephantulus myurus*; Order Macroscelidea; Figure 2) lose their zona pellucida already by the 2-cell stage, and from the 4-cell stage must have formed tight junctions as they have developed a cavity in their centre [4]. As the blastomeres divide they remain on the outside of this unilaminar embryo until the 120-cell stage when cell divisions originating in all areas of the embryo wall lead to rounded daughter cells that protrude towards the inside and develop long pseudopodia. These cells detach and eventually lose their pseudopodia and coalesce to form the inner cell mass [4,65]. The formation of a unilaminar vesicle is reminiscent of marsupials, the sister group of the eutherians, though in these animals the cells equivalent in fate to the eutherian ICM do not migrate to the inside. These comparisons suggest that the early differential loss of polarity (or differential absence of a gain in polarity) is the critical eutherian adaptation that inherently led to two distinguishable cell types (polar/epithelial versus apolar) that would consequentially be positioned in two territories (outside versus inside; Figure 3G). The mouse studies clearly demonstrate how changes in polarity can be coupled to lineage cues. From an evolutionary perspective, the early partitioning into inside and outside lineages enables early outer lineage-specific specialisations required for subsequent implantation. Secondly, the epithelialisation of the outer layer provides a protected environment (in the absence of an egg shell) for the further specialisation of the inner lineage.

### 3.5. Epithelialisation of the Outer Cells

The polarisation of outer cells combined with the upregulation of proteins required to form various junctions (tight junctions, adherent junctions, desmosomes, and gap junctions [66]), allows the formation of an epithelial layer. This layer becomes impermeable from the end of the morula stage [67]. The sealing of the epithelial layer is accomplished by F-Actin rings that form around microtubule-enriched apical domains to then expand toward cell-cell junctions, where they couple with neighbouring actin rings. The coupling involves tight junction molecules, such as ZO1 and adherent junction components including E-Cadherin and β-Catenin [68]. Once coupled, Myosin-II is attracted and provides a mechanical tensional force that is necessary for the “zipping up” of the Actin rings, which seals the embryo [68]. Once sealed, the activity of various outer-cell specific Na^+^/K^+^-ATPase dependent pumps in combination with Aquaporins leads to the directional flow of solutes and water into the interior of the embryo [69]. This results in the formation of the blastocyst cavity, a fluid filled cavity that marks the transition from the morula to the blastocyst stage.

Specifically in outer cells, ATP requirements rise as the energy hungry Na^+^/K^+^-ATPases begin pumping sodium ions into the emerging blastocyst cavity [70]. Secondly, from the morula stage all blastomeres require more energy (ATP) than at the early cleavage stages as cell divisions are accompanied with growth and therefore increased protein synthesis [71,72]. The higher energy demands are met by the consumption of glucose in addition to pyruvate. Interestingly, in mice inner cells convert the majority of the glucose substrate to lactate via the oxygen-independent, but energy-inefficient metabolic pathway of glycolysis [73]. The cytosolic NAD^+^ used during glycolysis is replenished (i) partly by reducing pyruvate to lactate which is then secreted; and (ii) partly by blastomeres taking up aspartate and converting it to malate (the Aspartate-Malate shuttle, or MAS), which is subsequently oxidised in the mitochondrion [11]. For outer cells, glycolysis does not provide sufficient energy for their higher demands and thus a significant fraction of the consumed glucose is, after conversion to pyruvate, transported into the mitochondria to undergo oxidative phosphorylation. Overall, oxygen consumption increases [74,75], particularly in the outer cells, and this creates free oxygen radicals. Some of the radicals are mopped up by blastomeres diverting glucose to the pentose phosphate pathway which results in the production of the antioxidant glutathione [11]. This is critical as excess free radicals created by increased oxidative phosphorylation (oxidative stress) leads to an arrest or impairment of development [76]. Intriguingly, TEAD4, which is one of the TEAD proteins interacting with YAP1 and thus involved in outer cell lineage specification, was shown to be essential for mediating the embryos balancing act between obtaining sufficient energy while not succumbing to oxidative stress damage [77]. While *Tead4* deficient embryos do not develop to the blastocyst stage in vivo, they are able to develop normally and activate lineage specific genes in vitro, but only under conditions in which oxidative stress is minimised. It is unclear how *Tead4* mediates this effect, but it is likely to be linked to its unique (among TEAD proteins) translocation to mitochondria [77]. Notably, expression of *TEAD4* predominantly in outer cells has been reported in rat, cattle, rhesus monkey and human blastocysts [78]. While knock-down of *TEAD4* in cattle embryos via siRNA did not result in increased susceptibility to oxidative damage [79], this evidence is inconclusive as residual TEAD4 message and protein may have sufficed to prevent developmental arrest.

In view of the observation that TEAD4 is also detected in marsupial cleavage stage embryos [80], it is clear that this protein mediates an ancestral function during mammalian early development. It will be interesting to see whether the prime (ancestral) function lies in protecting the future trophoblast cells from oxidative damage, incurred through the energy demands to generate the blastocyst cavity, or in specifying these very same cells via the TEAD family’s interactions with YAP1 to regulate lineage determination genes.

## 4. Establishing the First Lineages (the Blastocyst)

As the blastocyst cavity expands, the inner cells are pushed to one side of the embryo. Morphologically, two different cell types corresponding to the first two lineages are now visible. These are, firstly, the mesenchymal-like inner cells, now termed the inner cell mass/ICM, and secondly, the surrounding unicellular layer of epithelialised prospective trophoblast cells. The ICM will give rise to the epiblast and hypoblast (primitive endoderm) forming the embryo proper as well as numerous extraembryonic tissues. The outer epithelial layer will contribute cells only to the placenta [81,82]. It is trophoblast cells that establish the initial contacts with the maternal uterine epithelium, which leads to attachment and subsequently implantation of the conceptus. Interestingly, embryos of different mammalian species attach at widely disparate stages of their embryonic development ranging from 4-cell (elephant shrew); to late blastocyst stages, in which the epiblast has just begun to form (rodents, primates); to embryos that are already in the midst of neurulation and early somite formation (sheep, cattle, pigs); or have even reached limb bud stages (horses) [3]. Such alternate timing may impose different requirements on the relative speed at which the trophoblast has to commit to its fate [83].

### 4.1. The Timing of the First Lineage Commitments

Commitment is defined experimentally by the ability of cells to maintain their fate when placed in a different ectopic environment. Waddington famously pictured the analogy of a ball running down a slope containing forked valleys—once in a valley it is committed to that trajectory or fate as it can no longer reach parallel valleys due to the intermediate ridges [84].

In mice, detailed lineage tracking in unperturbed embryos revealed that the 16-cell embryo is the earliest stage exhibiting cell fate bias—but only blastomeres of the 32-cell embryo are lineage restricted, giving rise to either trophoblast or ICM, but not both [52]. Competency-probing aggregation experiments showed that the outer, prospective trophoblast cells of 16-cell (compacted morula stage, see Figure 2) embryos were not yet committed to that fate [85,86]. Once embryos had started to cavitate after the 32-cell stage (early blastocysts), commitment had occurred [87]. This is about two cell cycles, or one and-a-quarter days, before implantation at the 160-cell stage on Day 4.5 [88]. The interval between commitment and implantation provides time for differentiation of trophoblast to occur. Interestingly, mouse blastomeres from the inside of the embryo only commit to an *ICM* fate later, somewhere between mid (64-cell) and late blastocyst stages [87,89,90,91]. In human mid-blastocyst stage embryos (up to 45-cell), the outer cells, while fated to become trophoblast, are not committed, as shown by being able to form NANOG-expressing ICM when aggregated to each other. By late Day 5 (late/expanded blastocysts or >180 cells) they are committed [92]. Embryos implant at E7, >260-cell stage [88], which provides an interval of one and a half days between commitment and implantation, which corresponds to only one cell division. Commitment of cattle trophoblast cells was tested by sandwiching outer prospective trophoblast cells between younger uncommitted blastomeres and tracking their fate. This revealed no commitment even at the late blastocyst stage (Day 7), but trophoblast cells were committed by Day 14, a time point just prior to gastrulation [83]. Attachment to the uterus occurs only on Day 21 [93]. In this mammal, though, commitment is much delayed in relation to mice. It nevertheless precedes implantation, in this case by at least a week. In sum we can conclude that trophoblast lineage commitment is a prerequisite for implantation and that trophoblast commitment occurs at species-specific developmental time points that are related to the time point of implantation.

### 4.2. Setting up Stable Lineage Gene Regulatory Networks (GRNs)

Preceding fate commitment is lineage specification which is dictated via a cell’s gene regulatory network. With the advent of technologies allowing dozens of genes, or even the entire transcriptome, to be measured in single cells. In combination with dimensionality reduction methods, such as Principle Component Analysis (PCA) or Diffusion Maps (DM), it has been possible to graphically compare the GRNs among blastomeres of an embryo and from different embryos. Such analyses in human, monkey, mouse and cattle blastomeres [94,95,96,97,98] revealed that blastomeres between egg and morula stages separate according to developmental age, forming a distinct stage-dependent progression in a PCA or DM representation. Blastomeres from the same stage clustered together. However, from morula to blastocyst stages, the GRNs of individual blastomeres of the same stage segregated either into two populations, correlating to trophoblast and ICM cells, or into three populations mapping to trophoblast, epiblast and hypoblast cells.

How does lineage specification relate to the experimentally determined lineage commitment time points? In mice, specification and commitment follow each other closely. Trophoblast cells became committed, as well as transcriptionally distinct, from ICM cells, and uncommitted 16-cell blastomeres at the early blastocyst stage (32-cell). In contrast, uncommitted 32-cell ICM cells were already transcriptionally distinct, though they did show a closer resemblance to the GRN of uncommitted 16-cell blastomeres than did 32-cell trophoblasts [95]. The commitment of human trophoblast progenitors at late blastocyst stages [92] tracks a day after the emergence of their distinct GRN in early blastocysts [94,96,99]. In cattle, trophoblast cells begin to acquire a distinct GRN beyond the late blastocyst stage, and even at this stage (the latest examined) some 7% of blastomeres could not be assigned by their gene expression profile to either lineage [97]. Commitment had not occurred at this stage [83]. We can conclude that GRN lineage specification generally occurs before commitment, but can also occur simultaneously as seen for mouse trophoblast. Secondly, GRN distinction between trophoblast and ICM cells occurs at distinct stages in different mammals—for mouse and human in early blastocysts, in cynomolgus macaque monkeys at early to mid-blastocyst stages (E6-7, 75–200 cells) [98] and in cattle beyond the late blastocyst stage. However, the order in which the first three lineages become transcriptionally distinct is conserved. In mice, the segregation of the first two lineages (trophoblast and ICM) occurs one cell cycle before ICM-like cells resolve into epiblast and hypoblast lineages [95]. In humans, the initial reports suggested that all three lineages appear to arise simultaneously [96]—yet, subsequent reanalysis of the data and a wealth of novel data indicated that in both human and monkeys a cell cycle separates these events [94,98,99].

In view of the different timings of implantation, lineage specification and commitment as well as the prior events of embryonic genome activation driven by enhancers from species-specific transposable elements, how (dis)similar are the first lineage GRNs and the mechanisms of their establishment, and stabilisation? Up to morula stages, distinct gene-co-expression modules identified in humans were largely preserved in mice, though the timing differed [24]. Thereafter, upon lineage specification, both conserved and species-specific (non-conserved) networks could be detected when comparing lineage-GRNs of different mammals [98,100,101]. While global expression studies are ideal for identifying new lineage-specific marker genes, additional loss and gain of function experiments are required to determine the functional importance of candidate genes. Overwhelmingly, such in vivo experiments have been performed only in mice. However, our understanding of lineage networks has also benefited enormously from in vitro studies using embryo-derived stem cell populations representing cells of various lineages that have adapted to proliferate stably in the presence of particular culture media and components. It should though be born in mind that such cell populations (2-cell-like, naïve and primed mouse and human embryonic stem cells (ESC), induced pluripotent stem cells (iPSC), epiblast stem cells, mouse trophoblast (TS) and hypoblast (XEN) stem cells) only approximate their source cells as the embryonic environment differs from culture media, and rapidly changes over time (an exception being diapause).

### 4.3. The Trophoblast Lineage

Focusing only on transcription factors, as these are fundamental in setting up transcriptional programs, the earliest trophoblast-specific markers in mouse embryos are *Cdx2* and *Id2*, followed by *Eomes*, *Elf5*, *Gata2*, *Gata3*, *Tfap2a* and *Tfap2c* [95,101,102,103]. Of these eight factors, *ID2*, *ELF5* and *EOMES* are not expressed at all in human preimplantation embryos [100]. Similarly, in cattle and pigs, *ELF5* and *EOMES* are not expressed in trophoblast before the blastocyst has hatched [104,105,106]. While *ID2* is initially ubiquitously expressed in cattle embryos, at late blastocyst stage it is found in epiblast and hypoblast rather than trophoblast [97].

#### 4.3.1. CDX2

Notably though, unique CDX2 expression in preimplantation trophoblast is highly conserved, having been recorded in humans [107], monkeys [98], cattle [108], pigs [109], rabbits [110] and even in the opossum, a marsupial mammal [80]. The onset of CDX2 expression differs among species, suggesting that its ancestral function lies not in the specification, but rather the stabilisation/survival of the trophoblast lineage. This is borne out by loss-of-function experiments. In the mouse, loss of CDX2 prevented the upregulation of trophoblast-specific genes, and affected blastocyst hatching and trophoblast cell number, yet trophoblast was formed [111,112,113,114]. Similarly, in rhesus monkeys, cattle and pigs, CDX2 knock-down still allowed trophoblast formation, but led to proliferation and hatching defects and downregulation of trophoblast-specific genes [83,109,115,116,117].

In mice, *Cdx2* is activated at the 8-cell stage primarily via the YAP-TEAD4 pathway, with YAP1 activated, via a mechanism involving the apical domain of polarised blastomeres, or potentially via mechanosensing (see previous section). However, redundant mechanisms appear to exist. Firstly, mouse embryos lacking TEAD4 can still activate CDX2 under conditions where TEAD4’s unique metabolic functions are bypassed [77]. Potentially TEAD1 and/or TEAD2, which (i) are also expressed at the 8-cell stage [118]; (ii) have identical DNA-binding properties to TEAD4 [119]; and (iii) are able to interact with YAP [120], can compensate for TEAD4 under these conditions. Secondly, *Cdx2* activation in the mouse involves TFAP2c protein, which can directly regulate *Cdx2* transcription via an enhancer located in its first intron [121]. TFAP2c also affects components of the apical domain, thereby indirectly (via YAP) activating *Cdx2* [121]. Thirdly, the GATA3 transcription factor regulates *Cdx2* transcription via the same intronic *Cdx2* enhancer [122]. Fourthly, NOTCH signalling can co-operate with YAP in activating a second trophoblast enhancer (termed TEE), located upstream of the *Cdx2* gene [123].

Unlike the early, pre-morula activation of CDX2 in mice, CDX2 protein is seen in outer cells only from blastocyst stages in rabbit, pig, cattle and human. This is well after polarisation and differential YAP signaling: Suggesting that the key role of YAP activation in the onset of *CDX2* transcription may not be conserved. The gene networks activated by CDX2 similarly may be quite distinct in different mammals. For example, the mouse CDX2 targets *Eomes* and *Elf5*, critical for mouse trophoblast maintenance, are not expressed early on in numerous other mammals. Secondly, the downregulation of *OCT4* (which drives the ICM/pluripotency GRN) by CDX2 is seen in the mouse [112,113,124], but not in cattle [83]. On the other hand, activation of *BMP4* may be a more common function of this transcription factor. CDX2 binds to a trophoblast-enhancer of *Bmp4*, activating transcription in mouse trophoblast stem cells [125]. Furthermore, in pig blastocyst embryos, modulation of CDX2 levels affected *BMP4* transcription [109]. Thus, while CDX2 is likely to be a pan-mammalian master regulator of the trophoblast lineage and required for its maintenance, the mechanism of its activation as well as of its actions appear to have diverged among mammals.

#### 4.3.2. GATA2, GATA3

In mice, GATA2 and 3 are both expressed from cleavage stages in all cells, but become restricted to the outer, trophoblast cells in late blastocysts [102]. Similar to *Cdx2*, *Gata3* requires TEAD4 for its activation [126]. GATA2 and 3 are at least partially redundant, being required for activation of trophoblast specific genes such as *Cdx2* and the efficient formation of blastocysts and, at later stages, for the correct differentiation of trophoblast cells [102]. In cattle, there is in vitro evidence using a trophoblast cell line, that GATA2 and 3 may affect the expression of trophoblast genes including that coding for the ruminant pregnancy recognition signal, Interferon-tau [127]. Cattle and human GATA2 and 3 are expressed in the trophoblast of late blastocysts though GATA3 protein in both species could also be detected at lower levels in the presumptive hypoblast [96,97,100,128].

#### 4.3.3. TFAP2a, TFAP2c

The TFAP2 family of transcription factors have a key role in the specification and maintenance of the trophoblast lineage, based on their expression and knock-out phenotype in mouse embryos and trophoblast stem cells [129,130]. In mice TFAP2c becomes restricted to the trophoblast in early blastocysts and appears to be involved in lineage-specification via its effects on *Cdx2* expression, polarisation of outer cells and modulation of the HIPPO (YAP) signalling-pathway [121]. Notably though, TFAP2c is not trophoblast-specific in human, Cynomolgus monkey, cattle, and pig embryos, where it is also highly expressed in prospective epiblast cells [97,98,100,131], and has a later role in mesoderm and primordial germ cell specification [132]. In contrast, TFAP2a is specifically expressed in trophoblast cells in both mouse and cattle blastocyst embryos [95,97]. TFAP2a appears to have overlapping functions with TFAP2c [129], raising the possibility that different mammals may have substituted TFAP2a for TFAP2c as the key gene for trophoblast specification.

We can conclude firstly that current results point to a conserved trophoblast-lineage GRN dominated by CDX2, GATA2/3 and TFAP2a/c. Secondly, the initiation of this core network in mammals other than mice is still unclear. Thirdly, downstream targets of the trophoblast-GRN appear to have diverged, which is in line with the different implantation strategies and timings among mammals.

### 4.4. The Pluripotent Inner Cell Mass Lineage

The ICM gives rise to all cells of the conceptus with the exception of trophoblast [133] and thus the term “*pluripotent*” was coined to reflect this difference from the *totipotent* cleavage stage blastomeres. Morphologically and functionally, the ICM is a very transitory state in that its constituent cells rapidly assume either an epiblast or hypoblast identity [134]. In mice [95], humans [94,99] and Cynomolgus monkeys [98], the trophoblast:ICM split in GRN-identity precedes that between the ICM derivative lineages by about one division (Figure 3G,H). The transitory existence of the ICM may explain why the ICM state has not been captured in stem cell lineages. Embryonic stem cells (ESC), isolated from mouse ICM, were shown to be pluripotent [135], however their contribution to hypoblast was minimal [136]; indicating that functionally, mouse ESC represent epiblast as opposed to bipotential ICM. This was verified recently in detailed analyses of the derivation and GRNs of such ESC cells [101,137]. This potential of a cell to contribute to all three embryonic germ layers and the germline, but not to hypoblast, is now termed “naïve” pluripotency or “ground-state” [138]. Primate ESC cultured under the original “standard-ESC” conditions (in the presence of Activin and FGF) differ from mouse ESC in that they correspond to a late “primed” stage of epiblast development [98], resembling epiblast undergoing epithelialisation. From 2013, human “naïve” ESC of an early epiblast character have been derived [139,140], reviewed in [141].

### 4.5. The Mouse ICM-Epiblast Gene Regulatory Network

The regulatory network stably sustaining mouse naïve ESC has been refined to a core set of 12 factors, including the transcription factors: OCT4 (POU5f1), SOX2, NANOG, SALL4, KLF2, KLF4, ESRRB, GBX2, and TFCP2L1 [142]. Apart from GBX2, all of these transcription factors are expressed in compacted morulas, mid-blastocyst ICM and late-blastocyst epiblast [101]; suggesting a key role in ICM and epiblast lineage establishment and maintenance. This is supported by a wealth of data:

(1) In vivo knock-out experiments: OCT4-deficient embryos develop to the early blastocyst stage but the inner cells stop expressing some epiblast (however NANOG is upregulated) and hypoblast markers, and instead start expressing trophoblast markers. Subsequently, all ICM–derived tissue is lost [143,144]. SOX2, while not required for the initial specification of ICM and epiblast, is critical for maintaining epiblast identity, including continued *Oct4* and *Nanog* expression [49,145]. Loss of NANOG led to normal early E3.5 blastocysts, but subsequent loss of epiblast, with blastocyst ICM-culture outgrowths forming only hypoblast [146,147]. SALL4 is required for both ICM and hypoblast derivation [148]. Some of the factors though did not appear to be involved in lineage decisions in this in vivo functional assay: double knock-outs of the closely related KLF2 and 4 genes [149] or of TFCP2L1 [150], ESRRB [151] or GBX2 [152] led to no impairment of early development in mice.
(2) An early differential expression in inner cells: *Sox2*, *Nanog*, *Klf2* and *Essrb* are among the first genes seen to be uniquely expressed in inner cells of 16-24-cell morulas [95].
(3) Downregulation of the trophoblast GRN: For example, OCT4 [124] and NANOG [153] directly repress the key trophoblast gene *Cdx2*.
(4) Pluripotent reprogramming ability: Overexpression of a cocktail of genes has been shown to be able to reprogram somatic cells to a naïve pluripotent state (so called “induced pluripotent stem cells” or iPSC). The initial cocktail contained three of the core pluripotency factors—OCT4, SOX2, KLF4—as well as c-MYC [154], Subsequently c-MYC was shown to be dispensable, and NANOG and SALL4 to aid, in the derivation of iPSC [155,156].

### 4.6. Conservation of the ICM-Epiblast Pluripotency GRN

Is this mouse pluripotency/ICM/epiblast core transcription factor GRN conserved across mammals? Studies in primates, using naïve ESC and examining embryonic gene and protein expression, have highlighted differences, but also a large degree of conservation with mice. In marmoset and Cynomolgus monkeys, all mouse homologous naïve ESC core factors apart from KLF2 (and GBX2) are expressed in the epiblast lineage [98,101]. In humans, OCT4, SOX2, NANOG, SALL4, KLF4 and TFCP2L1 are expressed, but ESRRB and KLF2 (and GBX2) are not [100,139]. Instead of KLF2, humans and marmosets express KLF17 [100,101], which potentially may substitute for KLF2 in an analogous fashion to zebrafish, where KLF17 and KLF2 have partially redundant functions during embryogenesis [157]. Primates and rodents are Euarchontogliran species (Figure 1). The more distantly related cattle, which belong to the sister clade (Laurasiotheria), closely follow the primate pattern with *OCT4*, SOX2, NANOG, SALL4, and *KLF4* expressed predominantly in the epiblast lineage at late blastocyst stages, with *KLF2* absent and *ESRRB* more abundant in trophoblast cells [97]. In pigs, ESSRB is downregulated at the blastocyst stage though expressed in, and important for, the maintenance of pig induced pluripotent cells [158].

Hence the consensus at this stage is that, of the core pluripotency factors, GBX2 and KLF2 are mouse specific, while ESSRB expression is variable even between closely related species. The greater similarity in the expression of core naïve pluripotency transcription factors between primates and cattle, compared to mice, may explain the recent finding that chimera formation can be obtained by injecting naïve human ESC into cattle and pig, but not mouse blastocysts [159]. Notably, the OCT4-SOX2-NANOG triumvirate, the members of which have been shown to activate each other and co-regulate pluripotency targets as well as repress the trophoblast GRN in human primed ESC [160], have been detected specifically in the epiblast of all mammalian species examined, including in addition to the aforementioned species, rabbits [161], pigs [162] and two species of marsupials [80,163]. The key role of these three factors is underlined by the observation that homologs and/or paralogs are involved in the generation of the initial pluripotent embryonic ground state, not only in mammals, but also non-mammalian amniotes [164,165], and even non-amniotic vertebrates such as fish and amphibians [166,167]. The dual requirement for OCT4 and SOX2 is likely to stem from the fact that OCT4 heterodimerises well with SOX2, with the complex binding a unique set of targets that forms the most common motif associated with pluripotency target genes [168]. Secondly, OCT4 and SOX2 (as well as KLF4) appear to be “pioneering” factors. They are able to bind target sites even when these are embedded in epigenetically silenced chromatin, thus acting as the transcriptional pioneers of the ICM/epiblast (pluripotent) lineage [169,170]. The widespread use of NANOG for establishment of the pluripotency network may seem surprising considering its low sequence conservation outside its DNA-binding homeodomain (for example, mouse and human NANOG are only 54% identical). Remarkably, a 70 amino acid fragment comprising only the homeodomain (a quarter of the protein) was sufficient to induce naïve pluripotency in mouse cells [167]. This fragment, similar to non-mammalian NANOG proteins, does not contain the WD domain responsible for cooperative interactions with core pluripotency factors OCT4 and SALL4 [171], suggesting that a critical ancestral subset of NANOG interactions encased in its unique homeodomain has been co-opted to achieve mammalian pluripotency.

While non-mouse mammalian germline knockout models for genes have been rare, CRISPR technology is beginning to overcome this limitation and has recently allowed the introduction of deletions in the *OCT4* gene in all cells of about half the human embryos injected [172]. This study revealed an earlier role for OCT4 in human embryos compared to mouse embryos. Similar to mice, expression of OCT4 target genes, including epiblast and hypoblast markers was lost, but unlike mouse *Oct4* knockout embryos, *NANOG* expression was lost as well and trophoblast genes (including *CDX2* and *GATA2*) were downregulated instead of upregulated, with a concomitant failure to form and/or maintain blastocyst stage embryos. The human OCT4 loss-of-function phenotype resembles the CRISPR-induced cattle OCT4 knockout, achieved using somatic cell nuclear transfer embryos. These embryos, in which maternal OCT4 persisted to morula stages, initially switched on NANOG, but at blastocyst stages lost NANOG expression [173]. Other lineage markers for all three lineages were downregulated as in the human embryos. siRNA OCT4-depleted cattle embryos also exhibited reduced expression of the trophoblast gene *CDX2*, as well as of the OCT4 target gene *FGF4*, while *NANOG* expression was reduced, albeit not significantly [117].

The accessibility of (non-pioneering) transcription factors is modulated by epigenetic modifiers which affect the methylation state and chromatin state of DNA. Conversely, many transcription factors can affect the localised epigenetic state of DNA, via their ability to tether chromatin or DNA modifiers. Recently TET1 (a factor involved in active DNA demethylation) and THAP11/RONIN, a DNA binding factor recruiting epigenetic modifiers, were shown to be exclusively expressed in human ICM and, together with MCRS1 (a factor involved in chromatin remodelling) to be sufficient to reprogram fibroblasts into naïve ESC [94]. TET1 and THAP11 are also essential in mice for ICM development as proven by in vivo functional studies [174,175]. TET1 is also expressed in cattle blastocysts, but ubiquitously [97]. Other pluripotency-associated DNA binding factors that function predominantly in attracting epigenetic modifiers such as PRDM14, DPPA2 and DPPA4 are expressed specifically in the ICM and epiblast of mice, humans and marmosets [100,101]. In cattle, PRDM14 too is specific to the ICM/epiblast though DPPA2 is not [97]. However, most epigenetic modifiers (TET1 being an exception) do not show a lineage specific distribution [34], suggesting that epigenetic modifiers may have more of an indirect function in lineage network establishment.

It can be concluded that the ICM lineage leading to epiblast (naïve pluripotency) is highly conserved with mice showing some differences that may be linked to this species’ requirement to more rapidly fully separate the trophoblast from the ICM lineages to accommodate precocious implantation [83].

## 5. The Third Lineage (Hypoblast)

The third lineage to arise in the embryo is the hypoblast (termed primitive endoderm in mice). In most eutherian mammals, hypoblast progenitors arise within the inner cell mass and subsequently cover the inner surface of the epiblast to eventually line the entire surface of the blastocyst cavity. ICM cells in mouse [176], rabbit [161], human [107], marmoset [101] and cattle [177] embryos initially co-express epiblast and hypoblast markers, but at later blastocyst stages this expression resolves into a “salt and pepper” pattern. This is where hypoblast and epiblast progenitor cells, now identified via exclusive expression of one or the other lineage marker, are interspersed in a seemingly random fashion.

### 5.1. The Mouse Hypoblast Gene Regulatory Network (GRN)

In the mouse, there is a definite sequence of hypoblast lineage marker activation (and/or concurrent shut-down in the alternate epiblast lineage) of GATA6 (8-cell), followed by PDGFRA (16-cell), SOX17 (32-cell), GATA4 (58-cell) and SOX7 (>64-cell) [178,179]. Earlier markers are expressed more ubiquitously than later ones and, by the > 64-cell late-blastocyst stage, all become confined to ICM cells that have downregulated the initially ubiquitously expressed epiblast marker NANOG. GATA6 lies at the top of the hypoblast GRN network, in that loss of this gene prevents the activation of all subsequent hypoblast markers. Even only a mild reduction in GATA6 levels (in *Gata6*+/− heterozygous embryos) is sufficient to reduce the number of hypoblast progenitor cells, causing a delay in hypoblast specification [180,181]. Furthermore, *Gata6* overexpression is able to reprogram ES cells into hypoblast stem (“XEN”) cells [182]. GATA6 is initially found in all blastomeres, then becomes progressively restricted to the subset of ICM-cells that will form the hypoblast [178]. Hence the pertinent question regarding hypoblast lineage determination is how GATA6 expression is *maintained* in prospective hypoblast cells, while concomitantly shut down in prospective epiblast cells. This transition is asynchronous, occurring heterogeneously in individual cells between the early (32-cell) and late (120-cell) blastocyst stages, such that over time more and more cells have transited from a GATA6-NANOG double positive state to expressing one or the other marker [183]. Subjecting single positive cells to an alternate signalling environment did not change their fate, indicating that lineage commitment is achieved once an ICM cell expresses NANOG or GATA6 in a mutually exclusive fashion [183,184].

### 5.2. FGF Signalling in the Mouse Hypoblast/Epiblast Lineage Decision

The mutually exclusive GATA6/NANOG pattern appears to be established predominantly via cell-cell signalling mediated by FGF. FGF4 is necessary for the specification of the hypoblast, as shown in vivo by loss of function experiments of FGF4 [185,186], both receptors FGFR1 and FGFR2 [187,188], as well as the downstream component GRAB2, which is required for FGF-mediated RAS-MAPK signalling [176]. In all these mutant embryos, GATA6 expression is lost and all inner cell mass cells express NANOG to adopt an epiblast (naïve pluripotency) fate. Similarly, chemical inhibition of FGF receptors (with PD173074) or of the downstream kinases MAP2K1/2 (=ERK1/2; inhibited with PD0325901) could, in a reversible fashion, direct ICM cells to an epiblast-only fate if applied from the 32-cell early blastocyst stage [189], that is, before any cells show a reciprocal expression of GATA6 and NANOG. Conversely, exposure of embryos to exogenous FGF could direct NANOG/GATA6 double positive ICM cells to a hypoblast fate in a dosage dependent fashion [183,185,189]. This instructive role of FGF in directing ICM cells to a hypoblast fate is mediated predominantly by FGFR1 [187,188] and acts via GATA6, as FGF treatment could not rescue the hypoblast defect seen in *Gata6*−/− embryos [180,181].

*Fgf4* expression is under dual control by OCT4 and SOX2 [49,143,145,190,191]. While OCT4 is expressed more widely, SOX2 is the limiting factor, being restricted first to the ICM then to the epiblast. Thus, at the “salt and pepper” late-blastocyst stage, the SOX2/OCT4/(NANOG)-expressing epiblast progenitors are the predominant source of FGF4. These cells secrete FGF4 which mediates its effects in a paracrine fashion on the surrounding hypoblast progenitors, via both FGFR1 and the hypoblast-specific FGFR2 to activate/maintain the later hypoblast markers SOX17, PDGFRA and GATA4 [147,187,188]. However, at the 34–50 cell early blastocyst stage, when SOX2 (and OCT4) show no lineage-specific restriction *within* the ICM, differential *Fgf4* mRNA expression is already seen [95,192], and may be selectively inducing hypoblast differentiation in the most responsive surrounding cells. It is still unclear how this early differential expression in *Fgf4* expression arises. On the one hand, intrinsic stochastic fluctuation in expression levels could generate cells that by chance express either more FGF signal or a better response to FGF signalling, leading to respective biases toward the epiblast or hypoblast state. Such biases could subsequently be stabilised by signal reinforcement [192]. Alternatively, the bias in FGF signalling could be imparted by the history of the blastomeres. The reasoning is as follows. Inner apolar cells are not only generated from polar outside cells at the 8-cell stage, but also during the subsequent one or two rounds of outer cell divisions. It has been suggested that cells internalising at these later time points may be biased to form hypoblast [41]. Initial studies using lineage tracing of microinjected outer blastomeres did not support the hypothesis [189], but other studies using non-invasive tracing did find such a bias [193,194]. It could be argued that the later internalising cells had been subject to more YAP signalling, which prevents *Sox2* induction (see previous section; [49]), thus, resulting in a delay in FGF4 synthesis. Secondly, cells remaining on the outside may have accumulated more FGFR2 protein as this gene is progressively upregulated specifically in outer cells between the 16- and 32-cell morula stages [188,194]. Temporary retention of FGFR2 after internalisation may have sensitised these cells to FGF signals. As internalised cells are able to move within the ICM [189,193], such hypoblast-biased cells would disperse, creating a subsequent random salt and pepper pattern equivalent to the stochastic model. Interestingly, while in mice extended residency in the outer trophoblast-fated environment may bias inner cells toward the hypoblast lineage, a mechanism is less disputable in the distantly-related (Afrotherian) elephant shrew. In these mammals, which do not transit through a morula stage, hypoblast progenitors delaminate from the outer cells only *after* the ICM has formed and assemble on the ICM surface to directly form a distinct hypoblast layer (see panels 18–20 in [4]). It thus appears that their extended residency in the outer, future trophoblast-layer strongly biased them toward the hypoblast lineage allowing them to bypass a fate-refinement period within the ICM. In marsupials, hypoblast cells may also require extended exposure to a trophoblast-like environment in that the majority of hypoblast cells are seen to arise on the margin of the pluripotent (ICM-equivalent) disc, where they are in close proximity to the abutting trophoblast [163].

How the initial stochastic or ontogenic FGF-signalling induced bias in fate becomes stabilised in mice is not quite clear either. One mechanism may involve reciprocal negative feedback of *Gata6* and NANOG on each other’s transcription to amplify initially subtle differences in expression. In vivo evidence is that in NANOG-deficient embryos, GATA6 is upregulated [147] and in GATA6-deficient embryos, NANOG is upregulated [180,195]. In vitro results suggest that these effects may be direct, as NANOG can bind the *Gata6* enhancer in ESC cells to downregulate expression [196], whereas GATA6 overexpression in ESC cells downregulates *Nanog* within 12 hours of induction and GATA6 binding was enriched upstream of the *Nanog* gene [182]. A second mechanism may relate to different downstream effectors mediating the FGF response in epiblast and hypoblast progenitors, although the details are yet to be worked out [187,188].

### 5.3. A Common Hypoblast Gene Regulatory Network (GRN)

**Figure 4:**
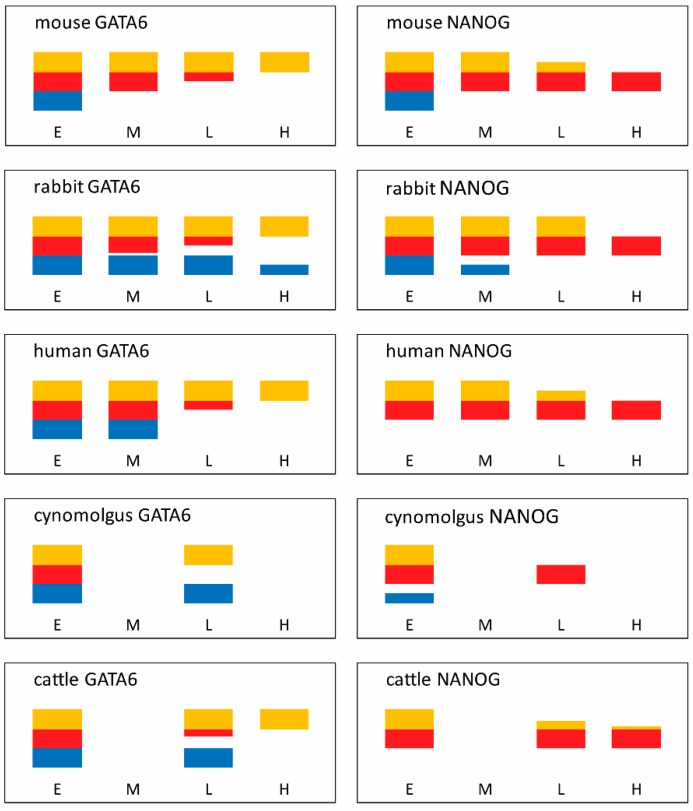
NANOG and GATA6 protein distribution in early (E), mid (M), late (L) and hatched blastocysts of various mammals. Lineages are colour coded: blue being trophoblast, red epiblast and yellow hypoblast. Blastocyst stages E, early, M, mid, L, late and H, hatched defined as per Table 2. Note extended expression of GATA6 in rabbit, old world monkey and cattle trophoblast at late blastocyst stages and extended maintenance of NANOG in rabbit prospective hypoblast at the late blastocyst stage. Based on data from the following references: mouse [49,178,179], rabbit [161], human [107,199,200], cynomolgus old world monkey [98] and cattle [177,201].

For the mouse, two key elements in hypoblast formation are FGF signalling and the central role of Gata6 at the top of the hypoblast GRN. How well are these elements conserved in mammals? Comparing the hypoblast GRNs, it appears that the sequentially activated and progressively refined mouse hypoblast markers GATA6, PDGFRA, SOX17, and GATA4 not only are all expressed in the hypoblast, but also appear in a similar temporal order in rabbit, human, old and new world monkeys, pig and cattle embryos (references as in Figure 4 as well as [100,101,197,198]). The progressive reciprocal restriction of expression of the epiblast (epi) markers NANOG and SOX2 relative to the hypoblast markers GATA6, SOX17, and GATA4 in mouse late blastocysts is also seen in the other eutherians at the late blastocyst stage, just prior to hatching (Table 2, Figure 4). GATA6 and NANOG have been examined in six species and invariably are detected ubiquitously in most cells from the earliest blastocyst stage, to then progressively resolve into hypoblast and epiblast domains respectively, with little to no overlap in expression upon blastocyst hatching (Figure 4). The eventual segregation of NANOG and GATA6 to epi and hypoblast lineages is even detected in marsupials [163]. There are though subtle differences in the regulation of GATA6 and NANOG. In the rabbit, the downregulation of NANOG in prospective hypoblast cells is delayed by one cell cycle, resulting in a transitory period where NANOG is still expressed in all ICM cells, whereas GATA6 downregulation in prospective epiblast cells has already commenced [161]. This suggests that if a direct inhibitory action of GATA6 and NANOG on each other’s transcription exists, it is likely not important for rabbits. Secondly, GATA6 expression is rapidly shut off in trophoblast cells in mice, but maintained to mid-blastocyst stages in humans and to late-blastocyst stages in rabbits, Cynomolgus monkeys, and cattle (Figure 4). The extended expression of the early-hypoblast marker GATA6 in the outer prospective trophoblast layer may well be indicative of an extended potential of these cells to give rise to hypoblast, in analogy to the elephant shrew mode of hypoblast formation discussed previously. Such extended fate plasticity has indeed been demonstrated for cattle [83].

### 5.4. FGF Signalling in Other Mammals

Therefore, it appears that overall the hypoblast regulatory network is very similar among all mammals examined to date. Is FGF signalling though universally involved in the establishment of this network? In rabbits, which are closely related to rodents, (i) exogenous FGF4 treatment transformed nearly all ICM cells to SOX17-expressing hypoblast progenitors and (ii), inhibition of FGF signalling via a MAP2k1/2 inhibitor prevented the formation of SOX17 positive hypoblast [161]. However, subtle differences existed: FGF pathway inhibition in rabbit embryos did not lead to an increase in the number of NANOG positive cells as in the mouse, but rather an increase in apoptosis. Secondly, GATA6 expression was not lost upon FGF pathway inhibition [161]. This difference to mice is more extreme in human embryos, which were shown to be refractory to FGF pathway inhibition (via FGFR- or MAP2K1/2-inhibitors) in regard to GATA6 and NANOG marker expression [177,199]. Marmoset monkey embryos responded uniquely to FGF-pathway inhibition by maintaining expression of GATA6, as well as the trophoblast marker CDX2, in most cells, while NANOG-expression was normal [101]. In the (Laurasiatherian) cattle and pig embryos, exogenous FGF could drive ICM conversion to GATA4/6-positive hypoblast as in mice and rabbits; but MAP2k1/2-inhibition resulted only in a partial conversion of hypoblast to epiblast progenitors as monitored via NANOG and GATA4/6 expression [177,198,203]. Interestingly, use of inhibitors targeting the FGF receptors as opposed to the downstream MAP2k1/2 had no effect on GATA4/6-expression in either species [177,198], but did affect the total number of ICM cells specifically in pig blastocysts [198]. This indicates a role for FGF signalling in pig ICM proliferation, but not in GATA6 maintenance.

When considering these gain and loss of function experiments, one must be consider that activation of hypoblast markers was achieved using extremely high concentrations of FGF4, usually 1000 ng/mL. This should be contrasted to the more physiological 25 ng/mL that is required to sustain FGF-dependent mouse trophoblast cells in culture [204]. Thus, hypoblast marker activation via exogenous FGF cannot be taken as proof that FGF4 is the endogenous lineage-determining ligand. This places more emphasis on the FGF pathway inhibitor results. In mice, use of such inhibitors led to a loss of GATA6 and downstream hypoblast gene expression, with all cells expressing epiblast markers such as NANOG and SOX2. As listed, this conversion to epiblast was at best only partially evident in the other mammalian species. The conclusion is that while FGF signalling may be sufficient for hypoblast specification, it is not necessary for this purpose in numerous mammalian species.

### 5.5. Alternative Signalling

The different phenotypes seen in various mammals when inhibiting FGF receptors (no effect) as opposed to the main downstream FGF signalling pathway (MAP2K1/2—some effects) could be explained by alternate signalling ligands and receptor tyrosine kinases that mediate their effects through MAP2K1/2. PDGFA is also able to signal through MAP2K1/2, however while PDGFRA is one of the earliest hypoblast markers, PDGF signalling is not involved in hypoblast specification in mice, but rather is required for prevention of apoptosis after establishment of the hypoblast lineage [205,206]. Furthermore, treatment of mouse embryos with exogenous PDGF-A (500 ng/mL) is unable to mimic the hypoblast-inducing effects seen with FGF [205]. Similarly, treatment of cultured mouse [207], human [208], cattle [209,210], or rabbit [211] embryos with other ligands able to activate MAP2K1/2 signalling, such as EGF and IGF1, have shown improvements in survival rates and increases in ICM cell number. However, these studies did not examine the hypoblast to epiblast ratio. Inhibiting most receptor tyrosine kinases (VEGFR, EGFR, PDGFR, FGFR) with the broad-spectrum inhibitor BI-BF1120 resulted in minimal changes in *NANOG* and *SOX17* and no effect on *SOX2* and *PDGFRA* mRNA expression in cattle embryos [212]. Hence in mammals other than mice and rabbits, the signals leading to MAP2K1/2 activation and the in vivo importance of such signalling in hypoblast specification are still unclear.

What about other signalling pathways? In cynomolgus monkeys *NODAL* is expressed in prospective epiblast cells [98]. In human embryos, genes of the NODAL/ACTIVIN/TGFβ pathway (the ligands NODAL and GDF3, the receptor ALK5/TGFBR1, NODAL-coreceptor TDGF1/CRIPTO, intracellular mediators SMAD2 and 4 and the target LEFTY1) are enriched in prospective epiblast cells at the late blastocyst stage [100]. Inhibition of this pathway using the ALK4/5/7 inhibitor SB431542 abolished NANOG expression indicative of a failure in epiblast establishment. However, expression of the hypoblast marker SOX17 was also lost in most embryos and levels of the ICM marker OCT4 reduced [100]. This argues for a role of NODAL-pathway signalling in human epiblast maintenance as opposed to hypoblast/epiblast lineage decisions. The lack of NODAL involvement in second lineage determination was also seen in marmoset monkey [101], cattle [177], and pig [198] embryos, where NODAL-pathway inhibition via either SB431542, or the even more potent A83-01 inhibitor, had no effect on the epiblast-to-hypoblast cell-number ratio. In mice, which differ from the other mammals by expressing NODAL only later, namely in mature epiblast (E5.5, corresponding to “primed” pluripotency) [98], SB431542 treatment had no effect on *Nodal*, *Sox17* or *Oct4* expression at late blastocyst stages [100]. Thus, in mammals, the role of NODAL signalling appears not to be involved in lineage decision, but may affect subsequent epiblast maintenance.

The third signalling pathway of potential influence is that of WNT. Canonical WNT signalling prevents β-Catenin degradation, allowing it to translocate to the nucleus to displace repressors from TCF/LEF sites so as to activate WNT target genes [213]. Among these target genes in naïve ESC are *Nanog*, *Klf2* and *Essrb* [214]. Activation of WNT-signalling by preventing β-Catenin degradation through inhibition of GSK3β with the chemical CHIR99021, is required for the maintenance of mouse and human pluripotent stem cells in vitro [215,216]. However, in mouse embryos it has been conclusively shown that WNT-signalling is only required from gastrulation stages onward and is dispensable during preimplantation and lineage determination [217]. In cattle, treatment with 3 µM CHIR99021 from zygote, but not from morula, stages resulted in slightly more NANOG cells with no change in the number of GATA6 positive hypoblast cells [177], indicating at most a minor effect of this pathway. Treatment of cattle embryos with DKK1 protein, which is an endogenous canonical WNT pathway inhibitor, resulted in a reduction in the number of blastocyst ICM cells, with a decrease in the epiblast (NANOG) to hypoblast (GATA6) cell ratio [201], suggesting that WNT signalling may be required specifically for prospective epiblast cell survival or proliferation. In marmoset monkey early blastocysts, the WNT signalling pathway may be specifically inhibited due to low expression of *β-Catenin* in combination with high expression of the WNT inhibitor *DKK1* and the β-Catenin destabiliser *GSK3*β genes (Boroviak15). This inhibition appeared to be relieved during ICM lineage-decision stages as DKK1 expression was diminished at the late blastocyst stage. Maintaining WNT signal inhibition via CHIR99021 from the early blastocyst led to a strong upregulation of the blastomere protein levels of NANOG, GATA6, as well as the trophectoderm marker CDX2 in late blastocysts. This pattern was similar to the coexpression of these markers seen before lineage segregation, at early blastocyst stages, leading the authors to infer a role for WNT signalling in marmoset ICM lineage determination (Boroviak15). In human embryos, functional data is not available, however *WNT3* mRNA increases sharply from early to late blastocyst stages, where it is restricted to the epiblast [99].

### 5.6. The Third Lineage—Conclusion

The picture that emerges from the available data is that the GRN driving the hypoblast lineage is well conserved across eutherian mammals, and may extend to marsupials as well. This GRN is driven by GATA6 with successive deployment of PDGFRA, SOX17, and GATA4 with concomitant exclusion of the key epiblast genes NANOG and SOX2, while OCT4 expression is maintained. However, the deployment of the hypoblast GRN has diverged in that FGF signalling is strongly implicated in the mouse and rabbit, but less so in primates and Laurasiatherians. No clear candidates substituting for FGF’s role have emerged. It may be that in these species the timing of internalization, and thus, the length of exposure in the trophoblast precursor environment, is important for generating the initial differences in ICM cells that will bias cells to either the hypoblast or epiblast lineage. I have discussed some data indicating that such a bias may also exist in mice, though its influence may be subjugated in this species by FGF dependent mechanisms.

## 6. Concluding Remarks

In summary, early mammalian development up to the hatched blastocyst stage is driven by a very limited set of building principles. The arguably most important ones can be listed as follows:

1. An intrinsic trigger to switch on the embryonic gene expression program. This trigger (DUX-C) is nearly fail-proof thanks to being present in the genome in high copy numbers.
2. The use of inherent random fluctuations (noise) in the gene expression machinery to generate asymmetries between blastomeres, which is likely to play a part in biasing cells during the first and second lineage decisions.
3. The adaptation of basic cellular processes (polarisation, compaction—as seen during mesenchymal to epithelial transitions) to asymmetrically segregate lineage specifiers during subsequent cell divisions.
4. Amplification of small differences in GRN-biases via reciprocal inhibition between alternative GRN programs. For the first lineage decision such inhibition is achieved predominantly through a small set of master transcriptional regulators, for the second decision additional control is achieved through the use of diffusible signalling molecules.

As already anticipated over 2000 years ago in Aristotle’s epigenesis idea, each step during the building of the blastocyst is dependent on the prior one—and as new lineages and distinct cell populations form, new avenues for autonomous interactions between these cell populations arise: Leading to the ever-increasing complexity of the mammalian embryo.

## Figures and Tables

**Table 1:** Glossary of embryological terms (in bold), alternatives often used (in regular font) in various species and their definition as used in this review.

Term and/or Abbreviation	Equivalent (Species)	Description
**conceptus**		Refers to all tissue derived from the zygote (embryonic and extraembryonic).
**embryo**	embryo proper	Before gastrulation equivalent to conceptus. From gastrulation stages refers to the embryonic parts of a conceptus that will give rise to the foetus as opposed to the extraembryonic membranes and placenta.
**Epiblast (epi)**		Derived from ICM, progenitor population of the three germ layers as well as the amniotic ectoderm and primordial germ cells.
**Hypoblast (HB)**	Primitive endoderm (mouse)	Cells differentiated from the ICM not contributing to the epiblast. The hypoblast will give rise to the inner layer of the yolk sac and, in primates, to extraembryonic mesenchyme.
**Inner cell mass (ICM)**	Pluriblast (marsupial)	Cells giving rise to epiblast and hypoblast.
primitive endoderm	**HB, Hypoblast**	Primitive endoderm is an alternative name to hypoblast and not to be confused with true (definitive) endoderm.
**Polar trophoblast (pTB)**	**Rauber’s Layer (e.g., cow, pig, rabbit, horse)**	Trophoblast overlying the ICM or epiblast.
**Trophoblast (TB)**	TE (mouse)	Extraembryonic layer: cells giving rise to the conceptus-derived part of the chorionic membrane and subsequently the foetal part of the placenta.
**Trophectoderm (TE)**	TB	During blastocyst stages, before overt differentiation, the trophoblast epithelium is often termed trophectoderm.

**Table 2:** Characteristics of early to hatched blastocyst stages in mammals ^1^.

Species:	Mouse	Rabbit	Human	Cynomolgus	Cattle
**Early** (cavity visible, <30% vol)	From 32 cells	From 64 cells	From 35 cells	From 50 cells	From 64 cells
	E3.25	E3 (“VI”)	E4–early E5	E5–6	E6
**Mid** (ca 30–70%)	<64 cells	>128 cells	64–100 cells		100–130 cells
	E3.5	E3.25 (“VII”)	late E5		E7
**Late** (max cavity zona enclosed)	>64 cells	>256 cells	128–256 cells	200–300 cells	140–200 cells
	E3.75	E3.5 (“VIII”)	early E6	E7-8	E7
**Hatched** (Hypo forming layer)	>100 cells	>512 cells	>256 cells	300–600 cells	>250 cells
	E4.25	E3.75 (“IX”)	late E6	E8-9	E8

^1^ Based on descriptions from references: mouse [178], rabbit [161], human [107], cynomolgus [98] and cattle [64,202].
